# Heart Dysfunction in Essential Hypertension Depends on Systemic Proinflammatory Influences: A Retrospective Clinical Pathophysiological Study

**DOI:** 10.3390/pathophysiology29030036

**Published:** 2022-08-07

**Authors:** Anton V. Barsukov, Alexander E. Korovin, Leonid P. Churilov, Ekaterina V. Borisova, Dmitry V. Tovpeko

**Affiliations:** 1 Kardio Klinika, 196105 St. Petersburg, Russia; 2 Laboratory of the Microangiopathic Mechanisms of Atherogenesis, Department of Pathology, Saint Petersburg State University, 199034 St. Petersburg, Russia; 3 S.M. Kirov Military Medical Academy, 194044 St. Petersburg, Russia; 4 Saint Petersburg Research Institute of Phthisiopulmonology, 194064 St. Petersburg, Russia

**Keywords:** essential arterial hypertension, chronic heart failure, preserved ejection fraction, diastolic dysfunction, proinflammatory status, 6 min walk test, correlation analysis, sex differences

## Abstract

Low-intensity systemic inflammation is an important element of heart failure pathogenesis. The aim of this study is to assess proinflammatory status serum indicators (C-reactive protein (CRP), tumor necrosis factor alpha (TNF-α), interleukin-6 (IL-6)) in middle-aged males (M) and females (F) with essential hypertension (HTN) depending on left ventricular (LV) diastolic dysfunction (LVDD). The main group comprised 55 M and 49 F with the first- to second-severity grade HTN with mild heart failure and a preserved LV ejection fraction ≥50%. Patients had sinus rhythm, first or second-severity degree LVDD, LV hypertrophy, left atrium dilatation, and NT-proBNP > 125 pg/mL. Comparison group: 30 hypertensives without cardiac dysfunction; control group: 31 normotensives. Quantitative features were compared using the Mann–Whitney test, median χ^2^, ANOVA module. Spearman’s rank correlation coefficients were determined to identify the relationship between the proinflammatory pattern and exercise tolerance. Hypertensive M had markedly higher CRP, TNF-α, and IL-6 levels compared to F. All mean values corresponded to reference range. In patients with second-degree LVDD, CRP, TNF-α, and IL-6 levels were significantly greater than in subjects with first-degree LVDD (both within M and within F samples). Significant negative associations between CRP, IL-6, and TNF-α levels and the 6 min walk test existed in hypertensive M and F. The study demonstrated a close relationship between the proinflammatory pattern and LVDD and exercise tolerance indicators, regardless of the hypertensive patient’s sex.

## 1. Introduction

Arterial essential hypertension (HTN) substantially occupies a leading position among population risk factors for heart failure in various regions of the world [1,2]. The formation of the so-called hypertensive heart is the basis of the pathogenesis for many common disorders of the left ventricular (LV) contractile and relaxive (diastolic) functions. It is considered that approximately half of all cases of chronic heart failure (CHF) occur while the status of LV systolic function is still normal [3]. The research has proved the dominant role of arterial hypertension in the occurrence of CHF with preserved LV ejection fraction (EF) (HFpEF) [4,5]. A typical phenotype of a patient with CHF, proceeding with normal LV systolic function, suggests a combination of: old age, female sex, arterial hypertension, obesity, type 2 diabetes mellitus, renal dysfunction, and a low level of physical activity [6]. The benefits of specific therapeutic approaches in patients with a similar variant of CHF have not been convincingly determined [7]. Nowadays, the importance of immune and inflammatory mechanisms in the pathogenesis of cardiovascular diseases is receiving strong factual support. One study demonstrated that HTN and other components of the metabolic syndrome are associated with systemic vasculitis, including autoimmune disorders [8,9]. Atherosclerosis and hypertension progressing are accelerated in patients with systemic autoimmune disorders accompanied by vasculitis syndrome [10]. There is a concept which regards vasculitis of vasa vasorum or small periadventitial vessels as a key link of atherogenesis and related cardiovascular disorders [11,12,13,14]. Excessive systemic action of proinflammatory autacoids may cause the development of CHF, based on the paradigm of systemic non-infectious low-intensity inflammation. This concept can be important not only for improving the understanding of this disease’s pathogenesis, but also for optimizing treatment decisions, although the influence of the systemic proinflammatory factors of heart function in HTN is still not clear. Thus, the aim of our study is to assess serum indicators of proinflammatory status as a correlate of immunopathological influences on cardiovascular system in male and female patients with essential hypertension depending on left ventricular diastolic dysfunction.

## 2. Materials and Methods

### 2.1. Object of the Study

**Figure 1:**
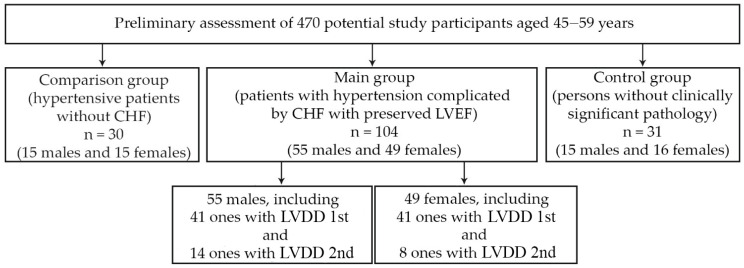
Scheme for the selection of the study participants in the main group (combination of essential arterial hypertension and heart failure with a preserved left ventricular ejection fraction). Abbreviations: CHF: chronic heart failure, LVEF: left ventricular ejection fraction, LVDD: left ventricular diastolic dysfunction.

This research was conducted with the participation of outpatients. The prescreening involved 470 units of medical documentation (outpatient records, case histories) of individuals aged 45–59 years. In total, 104 patients (55 males and 49 females) with essential HTN and HFpEF were selected for the main group. Thirty persons (15 males and 15 females) with essential HTN but without HF were chosen as a comparison group, and 31 people (15 males and 16 females) without any hypertension and other clinically significant disorders were designated as a control group. The main cohort of 104 specially selected patients (55 males and 49 females) had the diagnosis of essential HTN, grades 1 or 2 (n = 60 and 44, respectively) complicated by HFpEF. The scheme for the selection of study participants is shown in Figure 1. The individuals in the main group were selected according to the research design, representing a fairly homogeneous sample (on the basis of intragroup comparability by age, body mass index, severity of hypertension, heart failure, concomitant pathology). Males and females were initially comparable in age, body mass index (BMI), level of systolic (SBP) and diastolic (DBP) blood pressure, heart rate (HR) at rest, and severity of CHF. The diagnoses of essential HTN and HFpEF were established according to the current recommendations of European boards of experts [15,16]. The cohort included only patients with a clinical picture corresponding to a low functional class of CHF (the first or the second according to the NYHA classification), an LVEF equal to or greater than 50%, with a sinus rhythm, and plasma level of the N-terminal pro-brain natriuretic peptide (NT-proBNP) > 125 pg/mL. The NT-proBNP concentration was determined by the method of solid-phase enzyme immunoassay with Cobas H 232 Kit (Roche Diagnostics GmbH, Mannheim, Germany). The results of a 6 min test confirmed the functional CHF class. The notion of HFpEF was confirmed by the presence of the following criteria recommended by the European Society of Cardiology (2016): presence of LVH (proved by LV mass index (LVMI 115 (M) or >95 (F) g/m^2^)), dilatation of the left atrium (LA volume index > 34 mL/m^2^), and diastolic LV disturbances [15]. All individuals had the 1st or the 2nd stages of LV diastolic dysfunction (LVDD). Thus, the inclusion criteria were: age over 44 and under 60 years, essential HTN of the grade 1 and 2, HFpEF of the 1st or 2nd functional classes, NT-proBNP level > 125 pg/mL, and the presence of sinus rhythm. The following exclusion criteria were considered: age over 60 and under 44 years, the grade 3 of essential HTN, secondary arterial hypertension, history of myocardial infarction, non-coronary myocardial diseases, valvular heart diseases, atrial fibrillation and other clinically significant arrhythmias, chronic kidney disease of the fifth stage, any clinically significant pulmonary diseases, thyroid dysfunction, acute inflammatory diseases or exacerbations of any chronic disease within 2 weeks prior to screening day, as well as the presence of any oncological diseases. The study did not include patients with the inability to ensure the proper quality of echocardiography or the inability to assess diastolic LV function in sufficient capacity. All participants provided voluntary informed consent to take part in the study.

### 2.2. Laboratory Methods

The serum levels of the C-reactive protein (CRP), tumor necrosis factor alpha (TNF-α) and interleukin-6 (IL-6) were examined at 8–9 a.m. in fasting patients selected after the assessment of the inclusion/exclusion criteria. The CRP level in peripheral venous blood was measured by enzyme immunoassay with Sapphire-400 facility (Hirose Electronic System Co., Ltd., Tokyo, Japan), considering the normal level of less than 5.0 mg/L. The serum levels of TNF-α and IL-6 were assessed by means of a solid-phase enzyme-linked immunosorbent assay using highly sensitive test systems (Vector-Best, Novosibirsk, Russia). Biochemical blood analysis was performed with a spectrum analyzer (Abbott, Abbott Park, IL, USA). Reference ranges of C-reactive protein, TNF-α and IL-6 content were 0–4.9 mg/L, 0–5.9 pg/mL and 0–9.9 pg/mL, respectively.

Walking distance (in meters) with turns along a long straight corridor (30 m) was measured at the patient’s own pace in 6 min. The examined patients were allowed to slow down and stop during the test, but if possible, the walk was be resumed immediately, since the stopwatch was not stopped. A normal test result (indicating the absence of heart failure) was a distance of more than 525 m.

### 2.3. Instrumental Methods

Standard twelve-lead electrocardiograms were recorded with the ECG-9812 electrocardiograph (Medinova Industrial Co., Ltd., Shenzhen, China). Transthoracic echocardiography was performed according to standard procedures with the Philips EPIQ 7 ultrasound system (Philips Healthcare, Eindhoven, The Netherlands). Locating was performed at the chordal level of the mitral valve, directly below the free ends of its valves. Echocardiographic indices were evaluated in five subsequent cardiac cycles with the calculation of mean values. In this position, the LV internal diastolic dimension (LVIDD, mm), the LV internal systolic dimension (LVISD, mm), the interventricular septum thickness in diastole (IVS, mm), and the posterior wall thickness of the LV in diastole (PWT, mm) were examined. LV end-systolic volume (LVESV, mL), LV end-diastolic volume (LVEDV, mL), and LVEF were determined from the apical four-chamber and two-chamber positions by the disk method or by the modified Simpson’s method [17]. The LV mass index (LVMI, g/m^2^) was determined as the ratio of LV mass (LVM, g) to the body surface area (S, m^2^) using the following formula:

LVMI = LVM/S(1)

The LVM was calculated by the following cubic formula recommended by ASE experts (1986) [18]:

LVM, g = 0.8 × (1.04 × ((LVIDD + PWT + IVS)³ − LVIDD³) + 0.6(2)

Left ventricular geometry was estimated in accordance with the recommendations of the American Society of Echocardiography and the European Association of Cardiovascular Imaging (ASE/EACI, 2015) [19], which took into account the definition of LVMI and the relative wall thickness (RWT) of the LV. The RWT was calculated by the following formula (ASE/EACI, 2015):

RWT = (PWT + IVS)/LVIDD(3)

**Figure 2:**
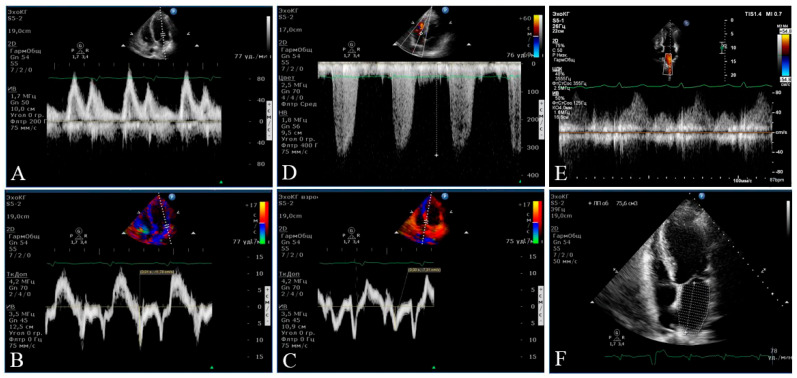
An episode of echocardiography in Participant B (female, 59 years old) with the 2nd stage of left ventricular diastolic dysfunction. In the Doppler mode (**A**), transmitral blood flow is shown with an assessment of Peaks E and A. In the tissue (**B**,**C**), the Doppler mode and the fibrous ring of the mitral valve movement are shown in the left ventricle early filling phase (Peaks e′ lateral and medial). (**D**) demonstrates the velocity of tricuspid regurgitation. (**E**) reflects the measurement of the flow in the pulmonary veins. The four-chamber position for measuring the volume of the left atrium is shown in (**F**).

LV diastolic function was examined in the mode of tissue Doppler echocardiography. The average peak tissue velocity of early diastolic displacement of the septal and lateral parts of the mitral valve ring (e′, cm/s) was estimated, and the E/e′ index was calculated to establish the left ventricular filling pressure [15]. An episode of ultrasound assessment of left ventricular diastolic function is shown in Figure 2.

The left atrium volume (LAV, mL) was measured by means of the area-length method in the B-mode using the formula:

LAV = 8/3π × A_1_ × A_2_/d (4)

where A_1_ is the area of the LA in the 4-chamber section, A_2_ is the area of the LA in the 2-chamber section, and d is the smallest size of the LA in the 2- or 4-chamber section (apical–basal).

The LA volume measured by the above-mentioned method was indexed by the body surface area (mL/m^2^), in accordance with the recommendations of ASE/EACI experts [20,21]. The normal LA volume index (LAVI) for the examined individuals was considered equal to or less than 34 mL/m².

### 2.4. Statistical Analysis

Statistical analysis of the data was performed using the Statistica software package for Microsoft Windows (StatSoft, Inc., Tulsa, OK, USA; software version 10.0.1011.0). Normal distribution was verified by the Kolmogorov–Smirnov method with the Lilliefors correction. The group differences were estimated by determining the value of the Student’s *t*-test, the relationship between the Pearson categorical variables (criterion χ^2^), and the ANOVA module. To study the relationships between indicators that did not obey the law of normal distribution, we used the module of non-parametric statistics (non-parametric Mann–Whitney U test). A critical level of significance was considered to be *p* < 0.05. Spearman’s rank correlation coefficients were calculated in order to identify the relationship between the proinflammatory pattern and exercise tolerance (the 6 min walk test). The correlation was considered to be weak for the coefficient r < 0.2; moderate for r = 0.2–0.49; and strong for r ≥ 0.5.

## 3. Results

The baseline characteristics of the examined patients with essential HTN complicated by HFpEF are summarized in Table 1.

According to our study, the level of each of the measured proinflammatory markers in the examined individuals with essential HTN and HFpEF corresponded to the reference range of values regardless of sex. Males and females had practically identical values for age, BMI, HbA1c, NT-proBNP, the 6 min walk test, systolic and diastolic BP, and heart rate (*p* > 0.05 for each parameter). The incidence of obesity, diabetes mellitus, and renal dysfunction was similar among males and females in the main group. Based on ultrasound data, both the male and female groups demonstrated a moderate concentric LVH with a slight left atrium dilatation, borderline changes (upward) of the E/e′ indicator, and a decrease in the e′ indicator, confirming the presence of non-severe LVDD. Males and females had no significant differences in parameters such as LVIDD, LVISD, LVEF, RWT, and LAVI (*p* > 0.05 for all parameters). LVMI and the E/e′ ratio in males proved to be significantly greater (*p* = 0.03) and the exponent e′ was significantly smaller (*p* = 0.009) than in females. However, the hypertensive males had markedly higher levels of CRP, TNF-α, and IL-6 compared to females (*p* = 0.001; *p* = 0.002; *p* = 0.001, respectively).

Table 2 and Table 3 shows the baseline characteristics in hypertensive patients without CHF and in examined healthy individuals, taking into account their gender.

As shown in Table 2, the levels of each of the measured proinflammatory markers, NT-proBNP, and 6 min walk test distance in the examined individuals with essential HTN without CHF corresponded to the normal values regardless of sex. Males and females had practically identical values for age, BMI, HbA1c, systolic and diastolic BP, and heart rate (*p* > 0.05 for each parameter). Based on ultrasound data, both male and female groups demonstrated a moderate concentric remodeling of LV without hypertrophy, normal left atrium size, and normal E/e′ and e′ indicators. Males and females had no significant differences in parameters such as LVMI, LVIDD, LVISD, LVEF, RWT, LAVI, E/e′ ratio, e′ (*p* > 0.05 for all parameters). The hypertensive males without CHF had similar levels of CRP, TNF-α, and IL-6 compared to females (*p* > 0.05 for all parameters).

As shown in Table 2, healthy individuals had normal and similar researched parameters (age, NT-proBNP, 6 min walk test distance, BMI, HbA1c, systolic and diastolic BP, heart rate, LVMI, LVIDD, LVISD, LVEF, RWT, LAVI, E/e′ ratio, e′, CRP, TNF-α and IL-6 (*p* > 0.05 for all parameters)) between males and females.

We evaluated the data obtained in males and females of the main group selectively, taking into account the peculiarities of their LV diastolic function disorders. According to the criteria in [22], the first stage of LVDD was determined in 41 males and 41 females, and the second stage was observed in 14 males and 8 females. The serum CRP level in males with the first stage of LVDD was significantly lower than that in males with the second stage of LVDD (*p* = 0.001), but it was significantly higher than in females with the first stage of LVDD (*p* = 0.001). The content of CRP in males with the second stage of LVDD was significantly higher than that in females with the second stage of LVDD (*p* = 0.012). In females with the first stage of LVDD, the level of CRP was significantly lower than in females with the second stage of LVDD (*p* = 0.003). The concentration of TNF-α in males with the first stage of LVDD was significantly inferior to that in males with the second stage of LVDD (*p* = 0.001), but significantly exceeded it in females with the first stage of LVDD (*p* = 0.005). The content of TNF-α in males with the second stage of LVDD was slightly higher than that in females with the second stage of LVDD (*p* = 0.06). In females with the first stage of LVDD, the level of TNF-α was significantly lower than in females with the second of LVDD (*p* = 0.001). The concentration of IL-6 in males with the first stage of LVDD was significantly inferior to that in males with the second stage of LVDD (*p* = 0.001), but it was significantly higher in females with the first stage of LVDD (*p* = 0.015). The content of IL-6 in males with the second stage of LVDD clearly exceeded that in females with the second stage of LVDD (*p* = 0.001). In females with the first stage of LVDD, the level of IL-6 was significantly lower than in females with the second stage of LVDD (*p* = 0.001). With regard to the serum levels of CRP, TNF-α and IL-6 in hypertensive individuals, each of these pro-inflammatory parameters in males significantly exceeded that of females (within the same stage of LVDD). In individuals with the second stage of LVDD, it significantly exceeded that of subjects with the first stage of LVDD (both for male and female samples).

**Figure 3:**
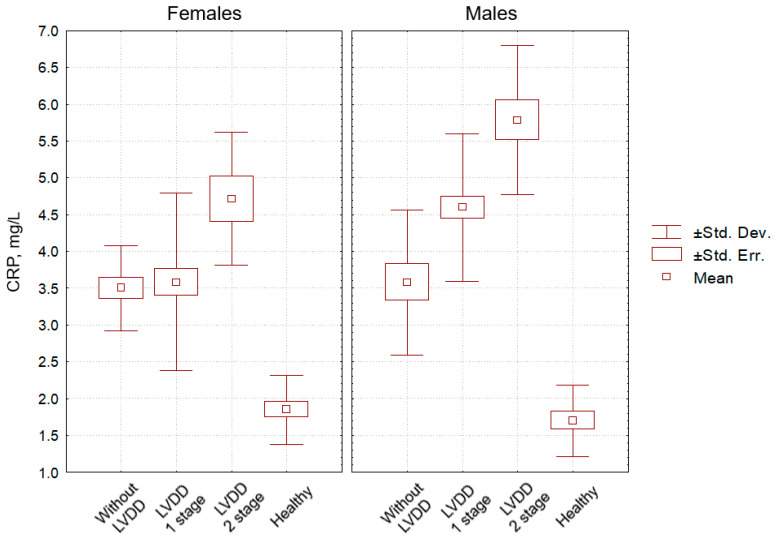
Indicators of CRP level in the blood (mg/L) in patients with hypertension without CHF and LV diastolic dysfunction (comparison group), patients with hypertension with CHF and LV diastolic dysfunction of the 1st and the 2nd stages, and healthy individuals (control group), taking into account participants’ gender.

**Figure 4:**
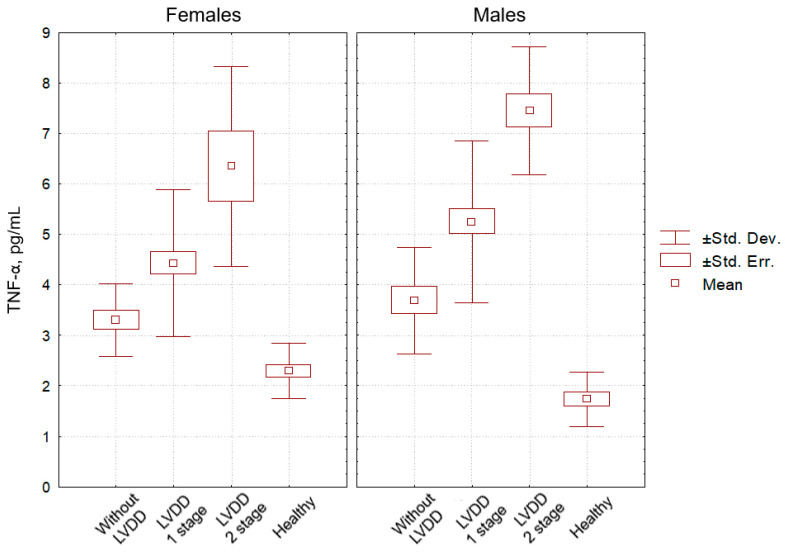
Indicators of TNF-α level in the blood (pg/mL) in patients with hypertension without CHF and LV diastolic dysfunction (comparison group), patients with hypertension with CHF and LV diastolic dysfunction of the 1st and the 2nd stages, and healthy individuals (control group), taking into account participants’ gender.

**Figure 5:**
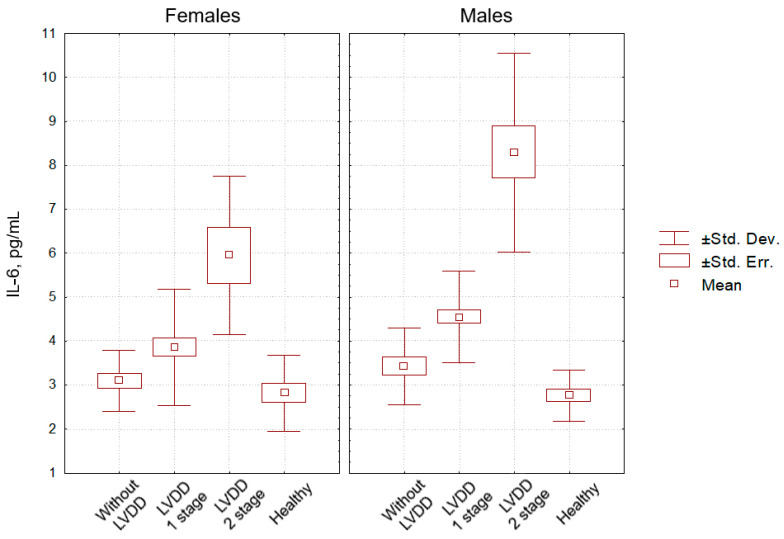
Indicators of IL-6 level in the blood (pg/mL) in patients with hypertension without CHF and LV diastolic dysfunction (comparison group), patients with hypertension with CHF and LV diastolic dysfunction of the 1st and the 2nd stages, and healthy individuals (control group), taking into account participants’ gender.

Figure 3, Figure 4 and Figure 5 clearly show the levels of CRP, TNF-α, and IL-6 in all categories of examined individuals, taking into account their gender, as well as the division of patients with CHF into subgroups depending on the degree of LVDD.

Both male and female patients with essential HTN complicated by HFpEF displayed significant negative correlations of the serum CRP, TNF-α, and IL-6 levels with the 6 min walk distance (males: r = −0.568, *p* < 0.001; r = −0.640, *p* < 0,001; r = −0.616; *p* < 0.001; females: r = −0.610, *p* < 0.001; r = −0.513, *p* < 0,001; r = −0.373; *p* = 0.001).

**Figure 6:**
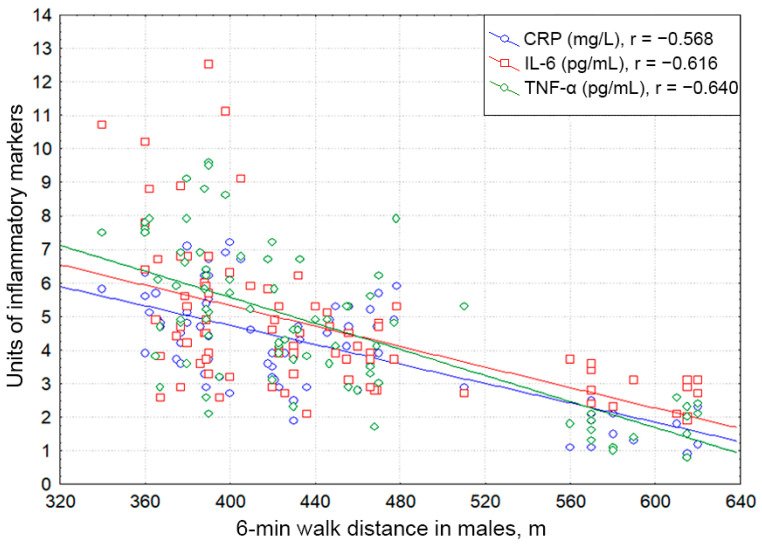
Correlative associations between C-reactive protein, interleukin-6, and tumor necrosis factor alpha serum levels and 6 min walk test results in males with hypertension complicated by CHF with preserved ejection fraction. Each dot represents a participant. Abbreviations: CRP: C-reactive protein, TNF-α: tumor necrosis factor alpha, IL-6: interleukin-6.

**Figure 7:**
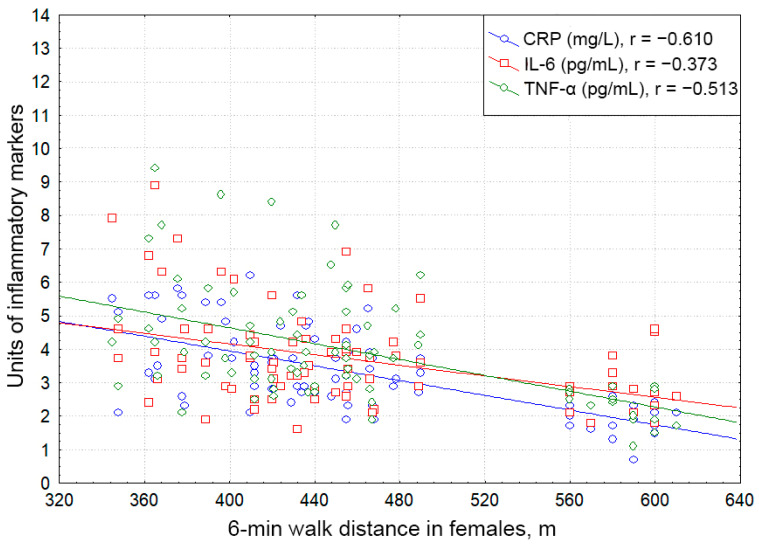
Correlative associations between C-reactive protein, interleukin-6, and tumor necrosis factor alpha serum levels and 6 min walk test results in females with hypertension complicated by CHF with preserved ejection fraction. Each dot represents a participant. Abbreviations: CRP: C-reactive protein, TNF-α: tumor necrosis factor alpha, IL-6: interleukin-6.

Figure 6 and Figure 7 depict the associations of proinflammatory mediators with exercise tolerance regarding gender.

The level of CRP in healthy males was significantly inferior to that in HNT males without LVDD (*p* = 0.03), with LVDD grade 1 (*p* = 0.01), with HNT and LVDD grade 2 (*p* = 0.001). The level of CRP in healthy females was significantly inferior to that in HNT females without LVDD (*p* = 0.05), with LVDD grade 1 (*p* = 0.05), with LVDD grade 2 (*p* = 0.01). In healthy men and women, as well as in hypertensive men and women without LVDD, the level of CRP did not differ significantly (*p* = 0.21 and *p* = 0.45, respectively).

The level of TNF-α in healthy males was the lowest, significantly inferior to that in HNT males without LVDD (*p* = 0.05), with LVDD grade 1 (*p* = 0.01), and with HNT and LVDD grade 2 (*p* = 0.001). The level of TNF-α in healthy females was significantly inferior to that in HNT females without LVDD (*p* = 0.05), with LVDD grade 1 (*p* = 0.03), and with LVDD grade 2 (*p* = 0.001). In healthy men and women, as well as in hypertensive men and women without LVDD, the level of TNF-α did not differ significantly (*p* = 0.11 and *p* = 0.09, respectively).

The level of IL-6 in healthy males was inferior to that in HNT males without LVDD (*p* = 0.10), with LVDD grade 1 (*p* = 0.05), and with HNT and LVDD grade 2 (*p* = 0.001). The level of CRP in healthy females was inferior to that in HNT females without LVDD (*p* = 0.12), with LVDD grade 1 (*p* = 0.07), and with LVDD grade 2 (*p* = 0.01). In healthy males and females, as well as in hypertensive males and females without LVDD, the level of CRP did not differ significantly (*p* = 0.88 and *p* = 0.75, respectively).

## 4. Discussion

The indicators of pro-inflammatory status (C-reactive protein, TNF-α, IL-6), corresponding to the normative range of values, were higher in males than in females both in uncomplicated hypertension and in the presence of HFpEF. The obtained gender differences in the level of the studied inflammatory biomarkers can be interpreted, to a certain extent, from the standpoint of differences in the indicators of LVDD, reflecting the severity of myocardial stress. The intramyocardial production of inflammatory mediators could influence their serum levels. It is possible that the revealed (insignificant) differences in the body mass index in males and females (a greater proportion of obese individuals among males than among females) can also contribute into the predominance of inflammation markers in the former. The addition of heart failure was associated with an additional increase in the level of C-reactive protein, TNF-α, and IL-6 in the blood, regardless of patients’ sex. The more severe (second) stage of LVDD was accompanied by higher systemic concentrations of proinflammatory autacoids compared to the initial (first) stage of LVDD, both in hypertensive males and females. Discussing the data obtained, one should point out the specificity of the sample examined. The patients in the main group included in the study were characterized by a mild to moderate increase in blood pressure (although most of them used various antihypertensive drugs), which was established in an office setting and corresponded to the study design.

The vast majority of surveyed males and females with CHF had abdominal obesity of alimentary-constitutional genesis of the first and second degrees, while some of them suffered from type 2 diabetes mellitus. In the given sample of examined individuals, HFpEF was verified on the basis of modern criteria. According to the study design, all patients had CHF due to essential HTN. Moreover, these features of the clinical portrait predetermined the specificity of their pro-inflammatory pattern relative to the comparison and control groups. We assessed the studied indicators, taking into account the comparability of the surveyed males and females provided for a number of signs. Thus, it should be emphasized that the interpretation of the data obtained cannot be performed solely from the standpoint of left ventricular dysfunction, but must take into account the entire complex of factors responsible for the pro-inflammatory pattern.

The majority of the study participants included in the main group regularly took antihypertensive drugs: 68% took ACE inhibitors or sartans, 56% took dihydropyridine calcium antagonists, and 37% took thiazide or thiazide-like diuretics. Along with this, in the interests of the primary prevention of cardiovascular diseases, 29% took antiplatelet agents, and 27% took statins. Overall, 10% of patients from the main group received metformin to correct carbohydrate metabolism disorders. Among the study participants included in the comparison group, 53% regularly took RAAS blocking drugs, 40% took dihydropyridine calcium antagonists, 20% took thiazide or thiazide-like diuretics, 17% took antiplatelet agents, and 17% took statins. In this project, we did not specifically study the effect of cardiovascular therapy on pro-inflammatory status, which may be considered as one of its limitations.

Indeed, over the past two decades, the pathogenetic and prognostic significance of inflammation, autoimmunity and systemic vasculitis in cardiovascular diseases has been actively discussed. It has been firmly proven that nonspecific low-grade inflammation observed in essential HTN and metabolic syndrome is associated with damage to target organs [9,23,24,25]. At the same time, convincing data about sex differences in the role of inflammatory mediators for HTN and CHF pathogenesis have still not been provided. In our research, the predominance of the studied indicators levels in males over those in females is traced clearly only in the presence of CHF with preserved LVEF. The inflammatory phenotype is intrinsic to concomitant conditions of hypertension (atherosclerosis, obesity, metabolic syndrome, diabetes mellitus, atrial fibrillation, and heart failure) [26,27,28]. In the implementation of the clinical manifestations of cardiovascular diseases, of great importance is the close pathogenetic alliance of the systemic and local action of inflammatory autacoids, oxidative stress, dyslipidemia, and endothelial dysfunction, which all interplay under the conditions of altered immunoneuroendocrine interactions.

The chronic systemic action of proinflammatory mediators in HTN is mechanistically related to abdominal obesity as an almost obligatory component of the metabolic syndrome. Visceral adipose tissue is an active endocrine organ producing resistin, leptin, adiponectin, ghrelin, extragonadal steroids with estrogenic activity, angiotensinogen, plasminogen activator-1 inhibitor, lipoprotein lipase, adipsin, retinol-binding protein-4, IL-6, TNF-α and other cytokines or adipokines [29,30].

A systemic excess of cytokines, which principally are not endocrine, but paracrine, autocrine and juxtacrine bioregulators of focal, short-distance action, as a rule, is associated with the failure of the barrier function of inflammation. This phenomenon causes a disequilibrium between local and systemic defensive mechanisms, which is always highly pathogenic, e.g., in acute situations, it is a common denominator and key mechanistic link of hemodynamic shock of various etiologies [31].

However, prolonged low-grade systemic proinflammatory status may be also highly pathogenic, causing chronic conflict of local and systemic defensive programs [32]. It reflects the severity of the clinical manifestations of such diseases as non-treated HTN, heart failure, type 2 diabetes mellitus, atrial fibrillation and their comorbidities [33,34].

It is believed that such cytokines as TNF-α, interferon-α, IL-1, IL-2, IL-6, IL-12, and IL-17 are endowed with numerous, mainly pro-inflammatory effects, but IL-4 and IL-10 demonstrate anti-inflammatory properties. However, these gradations are relative and depend on a permissive continuum. For example, IL-2 at the same time is a mandatory factor for survival and function of T-reg lymphocytes, which downregulate the intensity of immune response and inflammation [35]. Transforming growth factor beta, secreted in the early phase of the immune response, has anti-inflammatory properties; however, as the disease stabilizes, the same bioregulator promotes the progression of fibrosis [36,37,38].

In the last two decades, solid evidence has been obtained proving that the overexpression of proinflammatory cytokines plays an important role in the pathogenesis of CHF [39]. An unfavorable effect of immune activation inherent in patients with CHF with reduced LV contractility on survival during long-term follow-up was established. At the same time, it is known that individuals with HFpEF of this heart chamber, which are characterized by a less pronounced systemic excess of cytokines, are characterized by a slightly better, but, in general, unfavorable long-term prognosis [40].

Intracardiac and intravascular proinflammatory cytokines can enter the systemic circulation and exert some deleterious effects on the interaction of immune and neuroendocrine systems [32]. There may be created a vicious circle with the cytokine-derived activation of cell adhesion molecules and mononuclear cells, followed by the synthesis of a new pool of proinflammatory cytokines by the latter [41,42].

The inflammatory component concomitant local TNF-α expression is considered an important factor both in myocardial dysfunction and remodeling in CHF [43,44] and in atherogenesis as well [45]. Heart failure is characterized by one more vicious circle in which cytokines stimulate myofibroblasts, resulting in the acceleration of interstitial myocardial fibrosis production, thus worsening the diastolic function. Mechanical stress caused by LVDD stimulates the overproduction of chemokines, affecting the activity of inflammatory cells that complete the vicious circle.

With a slight increase in the intramyocardial and serum levels of proinflammatory cytokines (in particular, TNF-α and IL-6), which is inherent in HFpEF, as a rule, minimal interstitial myocardial infiltration is observed, with a slight to moderate overexpression of natriuretic peptides typical for LV hypertrophy and deterioration of LV diastolic function [46].

In this study, we revealed a virtually linear relationship between the systemic concentrations of the inflammatory mediators and degree of LV relaxant disorders or exercise tolerance. In our research, the serum levels of CRP, TNF-α, and IL-6 clearly depended on the severity of myocardial diastolic dysfunction, regardless of patients’ sex.

Moreover, similar findings were reported by other researchers as well. Thus, C.-K. Wu et al. (2011) documented in HfpEF patients the association of TNF-α and IL-6 blood levels with tissue Doppler parameters of LV diastolic function (a direct relationship with the E/e′ indicator and an opposite one with the e′ indicator) [47]. The authors noted that the ability of cardiomyocytes to synthesize TNF-α increases with increasing diastolic cardiac wall stress, i.e., with end-diastolic pressure in the LV cavity [47]. An increase in exercise tolerance was achieved by means of anti-interleukin therapy in patients with HFpEF who had elevated CRP. Moreover, according to the RELAX trial, in patients with HFpEF, high CRP levels were negatively associated with lower peak oxygen consumption during cardiopulmonary exercise test but not with the distance during the 6 min walk test [48].

A negative correlation between pro-inflammatory biomarkers and exercise tolerance has been noted in another studies; however, an increase in aerobic physical activity is accompanied by a decrease in the inflammatory pattern. The higher the level of CRP, TNF-alpha, IL-6, the lower the maximum oxygen consumption [49].

Our data on a more pronounced increase in the level of pro-inflammatory cytokines in both males and females as remodeling and diastolic dysfunction of the left ventricle progressed were reflected in another large studies. Thus, in particular, the AF-RISK study found that the expression of biomarkers of cardiovascular remodeling is more pronounced among males, and the expression of biomarkers of the metabolic syndrome is more pronounced among females [50].

## 5. Conclusions

1. Compared to females, males with essential hypertension complicated by HFpEF display significantly higher blood levels of pro-inflammatory autacoids: C-reactive protein, tumor necrosis factor alpha, and interleukin-6.
2. The formation of the clinical syndrome of hypertensive HFpEF is associated with an increase in the intensity of systemic pro-inflammatory pattern both among males and females.
3. Regardless of sex, hypertensive patients with the second stage of the left ventricular diastolic dysfunction have significantly higher (compared to the first stage) systemic blood concentrations of C-reactive protein, tumor necrosis factor alpha, and interleukin-6.

## Figures and Tables

**Table 1:** The baseline characteristics of the examined patients with essential HTN complicated by HFpEF (M ± std. d.).

Parameters	Males (n = 55)	Females (n = 49)	*p* Value
Age, years	53.7 ± 6.4	51.5 ± 6.2	0.19
BMI, kg/m²	33.6 ± 5.2	30.3 ± 3.6	0.12
HbA1c, %	6.08 ± 0.09	5.92 ± 0.08	0.39
NT-proBNP, pg/mL	287 ± 96.9	282 ± 105	0.84
The 6 min walk test, m	407 ± 74	420 ± 80	0.14
Office SBP, mmHg	156 ± 8.8	148 ± 6.4	0.18
Office DBP, mmHg	90.2 ± 4.4	85.5 ± 5.7	0.32
Heart rate, bpm	74.3 ± 12.5	76.1 ± 8.7	0.21
Ejection fraction, %	62.8 ± 0.68	61.6 ± 0.73	0.27
LVIDD, mm	50.1 ± 6.61	49.6 ± 7.12	0.64
LVISD, mm	33.2 ± 5.7	33.6 ± 6.24	0.69
RWT	0.49 ± 0.07	0.49 ± 0.06	0.24
LVMI, g/m^2^	128 ± 1.52	121 ± 1.17	0.03
LAVI, mL/m^2^	36.7 ± 0.36	36.1 ± 0.34	0.24
E/e′	9.85 ± 0.40	8.63 ± 0.38	0.03
e′ averaged, cm/s	7.41 ± 0.23	8.31 ± 0.24	0.009
CRP, mg/L (reference ranges: 0–4.9)	4.91 ± 0.15	3.77 ± 0.17	0.001
TNF-α, pg/mL (reference ranges: 0–5.9)	5.81 ± 0.24	4.74 ± 0.29	0.002
IL-6, pg/mL (reference ranges: 0–9.9)	5.52 ± 0.29	4.21 ± 0.22	0.001

Abbreviations: BMI: body mass index, HbA1c: glycosylated hemoglobin, NT-proBNP: N-terminal pro-brain natriuretic peptide, SBP: systolic blood pressure, DBP: diastolic blood pressure, LVMI: left ventricular mass index, LAVI: left atrium volume index, CRP: C-reactive protein, TNF-α: tumor necrosis factor alpha, IL-6: interleukin-6.

**Table 2:** The baseline characteristics of the examined hypertensive patients without CHF and LVDD (M ± std. d.).

Parameters	Males (n = 15)	Females (n = 15)	*p* Value
Age, years	50.4 ± 5.3	49.8 ± 6.7	0.88
BMI, kg/m^2^	28.6 ± 3.4	29.2 ± 3.1	0.78
HbA1c, %	5.42 ± 0.96	5.09 ± 0.88	0.15
NT-proBNP, pg/mL	79.2 ± 5.47	69.6 ± 6.20	0.65
The 6 min walk test, m	886 ± 76.4	812 ± 92.3	0.75
Office SBP, mmHg	142.5 ± 10.3	145.3 ± 9.8	0.48
Office DBP, mmHg	90.7 ± 12.5	92.4 ± 10.4	0.40
Heart rate, bpm	72.4 ± 6.3	76.3 ± 8.7	0.55
Ejection fraction, %	64.1 ± 4.18	65.6 ± 3.34	0.66
LVIDD, mm	49.4 ± 4.67	51.7 ± 5.49	0.56
LVISD, mm	32.7 ± 3.93	34.6 ± 5.14	0.71
RWT	0.48 ± 0.04	0.50 ± 0.07	0.16
LVMI, g/m^2^	122 ± 3.85	105 ± 7.14	0.01
LAVI, mL/m^2^	29.2 ± 1.47	27.6 ± 2.20	0.05
E/e′	6.66 ± 0.61	6.46 ± 0.58	0.17
e′ averaged, cm/s	11.8 ± 1.37	12.1 ± 1.09	0.19
CRP, mg/L (reference ranges: 0–4.9)	3.58 ± 0.98	3.42 ± 1.02	0.09
TNF-α, pg/mL (reference ranges: 0–5.9)	3.68 ± 1.06	3.33 ± 0.77	0.12
IL-6, pg/mL (reference ranges: 0–9.9)	3.42 ± 0.86	3.09 ± 0.68	0.10

Abbreviations: BMI: body mass index, HbA1c: glycosylated hemoglobin, NT-proBNP: N-terminal pro-brain natriuretic peptide, SBP: systolic blood pressure, DBP: diastolic blood pressure, LVMI: left ventricular mass index, LAVI: left atrium volume index, CRP: C-reactive protein, TNF-α: tumor necrosis factor alpha, IL-6: interleukin-6.

**Table 3:** The baseline characteristics of the examined healthy individuals (M ± std. d.).

Parameters	Males (n = 15)	Females (n = 16)	*p* Value
Age, years	51.3 ± 4.2	50.1 ± 4.5	0.47
BMI, kg/m^2^	24.6 ± 1.7	25.2 ± 1.8	0.16
HbA1c, %	5.1 ± 1.1	5.2 ± 0.8	0.35
NT-proBNP, pg/mL	35.7 ± 6.1	40.6 ± 8.2	0.78
The 6 min walk test, m	911 ± 102	895 ± 98.3	0.87
Office SBP, mmHg	120.5 ± 7.6	117.1 ± 12.5	0.55
Office DBP, mmHg	75.7 ± 5.1	70.5 ± 7.2	0.64
Heart rate, bpm	66.1 ± 7.9	70.5 ± 6.7	0.27
Ejection fraction, %	63.3 ± 3.98	64.5 ± 3.52	0.44
LVIDD, mm	49.3 ± 5.32	50.7 ± 3.41	0.29
LVISD, mm	33.3 ± 4.77	34.8 ± 3.50	0.38
RWT	0.47±0.04	0.49 ± 0.05	0.22
LVMI, g/m^2^	98.9 ± 6.26	91.1 ± 3.31	0.06
LAVI, mL/m^2^	26.7 ± 2.47	26.2 ± 1.94	0.22
E/e′	6.4 ± 0.63	5.9 ± 0.49	0.32
e′ averaged, cm/s	11.7 ± 1.03	11.9 ± 1.12	0.66
CRP, mg/L (reference ranges: 0–4.9)	1.95 ± 1.72	1.85 ± 1.42	0.10
TNF-α, pg/mL (reference ranges: 0–5.9)	2.43 ± 1.53	2.29 ± 1.55	0.21
IL-6, pg/mL (reference ranges: 0–9.9)	2.76 ± 1.07	2.81 ± 1.01	0.79

Abbreviations: BMI: body mass index, HbA1c: glycosylated hemoglobin, NT-proBNP: N-terminal pro-brain natriuretic peptide, SBP: systolic blood pressure, DBP: diastolic blood pressure, LVMI: left ventricular mass index, LAVI: left atrium volume index, CRP: C-reactive protein, TNF-α: tumor necrosis factor alpha, IL-6: interleukin-6.

## Data Availability

Not applicable.
